# Molecular Diagnostics of *Cryptococcus* spp. and Immunomics of Cryptococcosis-Associated Immune Reconstitution Inflammatory Syndrome

**DOI:** 10.3390/diseases12050101

**Published:** 2024-05-13

**Authors:** Irina Vlasova-St. Louis, Hesham Mohei

**Affiliations:** 1 GenoHealth LLC, El Paso, TX 79912, USA; 2 Department of Pathology and Laboratory Medicine, University of Pennsylvania, Philadelphia, PA 19104, USA; hesham.mohei@pennmedicine.upenn.edu

**Keywords:** molecular genetics testing, cryptococcal immune reconstitution inflammatory syndrome (C-IRIS), HIV, CM-IRIS, cellulomics, proteomics, genomics, transcriptomics

## Abstract

Cryptococcal infection poses a significant global public health challenge, particularly in regions near the equator. In this review, we offer a succinct exploration of the *Cryptococcus* spp. genome and various molecular typing methods to assess the burden and genetic diversity of cryptococcal pathogens in the environment and clinical isolates. We delve into a detailed discussion on the molecular pathogenesis and diagnosis of immune reconstitution inflammatory syndrome (IRIS) associated with cryptococcosis, with a specific emphasis on cryptococcal meningitis IRIS (CM-IRIS). Our examination includes the recent literature on CM-IRIS, covering host cellulomics, proteomics, transcriptomics, and genomics.

## 1. Introduction

Microbial and human genomics, proteomics, transcriptomics, and metabolomics are major branches of the expanding multiomics field. Infectious disease physicians, scientists, and public health professionals rely now on high-throughput technologies like next-generation sequencing in assessing host–pathogen interactions at multiple levels [1]. The recently ended COVID-19 pandemic has set the stage for public health microbial surveillance programs by whole-genome sequencing (aside from SARS-CoV-2) [2]. Molecular fungal surveillance is crucial for public health and the environmental management of invasive fungal species [3]. It enhances our ability to detect and respond to fungal infections, track antifungal resistance, and monitor the overall dynamics of fungal populations in diverse ecosystems [4]. The integration of molecular techniques with epidemiological and clinical data provides a comprehensive approach to understanding and mitigating the impact of fungal pathogens, particularly *Cryptococcus* spp. [4].

On the host side, there has been great interest in the identification of systemic and localized biomarkers for the early detection of cryptococcal infection, examining gene expression, protein abundance, and metabolite concentrations to uncover specific patterns associated with the course of cryptococcal disease. Additionally, understanding longitudinal changes in host immune cell function and cytokine profiles and the interplay between different components of the human immune system can enhance the accuracy, reliability, and prognostic value in connecting the cryptococcal burden with the disease course and potential complications [5]. One feature of the cryptococcal antigenic burden that complicates HIV infection is immune reconstitution inflammatory syndrome (IRIS). There has been great interest in the identification of systemic and localized biomarkers of IRIS at the level of multiomics, which is discussed in this review [6].

## 2. Genotyping of *Cryptococcus* spp.

*Cryptococcus* spp. are polyphyletic fungi that received global attention after the emergence of HIV/AIDS as the leading cause of morbidity and mortality, with a global incidence of over twenty-three million cases per year [7,8]. For developing countries with limited resources (e.g., Sub-Saharan Africa), cryptococcosis is the second most common cause of hospitalization and death after tuberculosis, and both are highly associated with HIV infection [9,10,11,12,13]. In response to the increased attention and understanding of the importance of Cryptococcosis, numerous research groups have undertaken the task of deciphering the genomes of *Cryptococcus* spp. The genomes of *Cryptococcus* spp. have been successfully sequenced, and this genomic information is accessible through various bioinformatics browsers, including www.genome.uscs.edu (accessed on 1 February 2024, Figure 1). Access to genomic data provides researchers and the scientific community with valuable insights into the fungal molecular and genetic aspects, aiding in the development of better diagnostic tools and preventive measures.

Five molecular variants have been recognized for *C. neoformans* isolates (VNI, VNII, VNB, VNIII, and VNIV) and four for *C. gattii* isolates (VGI, VGII, VGIII, and VGIV) [14]. The global molecular genotyping of *Cryptococcus* spp. determined VNI as a prevalent molecular variant worldwide. It was shown that the specific *Cryptococcus* lineages evolved over time to modulate host immune responses and alter their recognition by the innate immune system and, thus, exhibit pathogenicity [15]. Genetic variations of clinically important *Cryptococcus* isolates from individuals affected by HIV revealed hundreds of polymorphic variants in sequence type 93 that were associated with the fungal fitness effect [16]. A genome-wide association study of isolates from a similar cohort of patients as above demonstrated the significant association of particular gene variants (single-nucleotide variants or larger chromosomal alterations) with cryptococcal virulence, the CSF fungal burden, and the in vitro fungal growth rate attributed to sugar metabolism [17].

Cryptococcal transcriptomes exhibit particular changes in the cerebrospinal fluid of affected individuals. For example, the overexpression of the group of *Cryptococcus* genes within virulence/survival (cell wall) and metabolic pathways (glucose, fatty acids, and iron uptake) was identified in patients and also corroborated in animal models [18,19].

The next-generation sequencing of *Cryptococcus* spp. is currently beginning to evolve and is primarily used in research settings [20,21]. However, as public health laboratories are now well equipped with high-throughput next-generation screening instruments, methodologies for the environmental and clinical detection of the cryptococcal genome are developing rapidly [22]. Newly discovered mutations in cryptococcal DNA from clinical isolates alter differential drug susceptibility and may increase the virulence of *Cryptococcus* spp., which causes ineffective treatment outcomes and increases the risk of immune reconstitution inflammatory syndrome (IRIS) [23].

Great advances have been made in the diagnostic procedures of cryptococcosis. The FDA-approved nested multiplex PCR panel (meningitis/encephalitis FilmArray, BioFire Diagnostics, USA) is designed to differentiate the *C. neoformans* and *C. gattii* genomes (among other pathogens that cause meningitis) using the *CAP59* gene [24,25,26]. Multi-locus sequence typing (MLST) enabled the identification of 13 known sequence types (ST) and one novel one (ST number 93) in 75% of the clinical isolates of *Cryptococcus neoformans* variant *grubii* obtained from HIV patients in Southeastern Brazil [27].

Loop-mediated isothermal amplification (LAMP) is a point-of-care isothermal molecular testing method that has significantly improved the diagnosis of cryptococcal disease. LAMP is designed to identify the species of *Cryptococci* from clinical isolates targeting genotypes VNI, VNII, and VNIII of *C. neoformans* [28].

Amplified fragment length polymorphism (AFLP) is a DNA fingerprinting technique that combines PCR with restriction enzyme digestion. This method is popular in the examination and differentiation between cryptococcal genotypes. Combined with mating assays that were performed to determine the mating types of isolates of *Cryptococcus gattii* recovered from an outbreak on Vancouver Island, AFLP led to the identification of rare mating-competent isolates [29]. One Denmark study reported the combination of AFLP with multilocus sequence typing (MLST of nuclear loci including *LAC1*, *CAP59*, *IGS1*, *PLB1*, *URA5*, *SOD1*, and *GPD1*). Selected *Cryptococcus* isolates subsequently underwent antifungal susceptibility testing and a comparison with isolates across the world [30]. Similarly, the AFLP and MLST methods were utilized for subsequent multilocus microsatellite typing (MLMT) and phylogenetic analysis. This combination, along with antifungal susceptibility profiles, was determined using a phenotypic methodology [31]. Interestingly, this study compared *C. neoformans*/*C. gattii* from antifungal-resistant isolates from HIV-positive and HIV-negative patients and did not find any significant types of *Cryptococcus* spp. [31]. The AFLP method is slowly being replaced by long-read third-generation sequencing, which proves to be more cost-effective, even in resource-limited settings, to provide a genomic link between environmental and clinical isolates of *Cryptococcus* spp. cross-breeds [32]. 

Another high-complexity clinical test is the polymerase chain reaction and restriction fragment length polymorphism (PCR-RFLP). A recent Brazilian study utilized PCR-RFLP of the URA5 gene for the molecular typing of *Cryptococcus* spp. from clinical isolates [33]. Subsequent assessments of antifungal drug resistance and virulence factors allowed clinicians to assess individual *Cryptococcus* types’ potential to cause severe infections and propose appropriate management strategies [33]. Novel hypermutator strains have been identified by PCR-RFLP among *Cryptococcus* spp., conferring resistance to almost all available antifungal drugs, including fluconazole, flucytosine, rapamycin, amphotericin B, and FK506, thus reducing cryptococcal clearance (reviewed in [34]).

Various known and novel mutations have been identified by next-generation sequencing, including in the *FUR1* gene, which conferred resistance in ST5 isolates of *C. neoformans*; however, there were no differences found in *Cryptococcus* spp.’s phylogenetic diversity between patient groups [35]. Although, several molecular genetic techniques are currently available to differentiate pathogenic cryptococcal genes from related fungal species, the mutations responsible for drug resistance and their effects on the immunodeficient host response are not well understood [6].

Cryptococcal antigens play significant roles in the immunocompromised host immune response. Untreated HIV patients and individuals simultaneously affected by *Cryptococcus* are known to have a large cryptococcal burden in various organs, including the lungs and brain [36]. These individuals are susceptible to the adverse event known as immune reconstitution inflammatory syndrome (IRIS), which is discussed below.

## 3. Cryptococcosis-Associated IRIS

Immune reconstitution inflammatory syndrome associated with cryptococcosis (C-IRIS) is a complication that can occur in HIV/AIDS patients after the initiation of antiretroviral therapy (ART). In general, this phenomenon is observed with the rapid restoration of immune function on ART regimens. As the immune system strengthens, it can overreact to latent infections, causing inflammation and revealing symptoms of an excessive host inflammatory response against an antigenic burden (or live pathogen), including *Cryptococcus*-related burdens [36]. Cryptococcal meningitis is one of the opportunistic infections that can trigger IRIS when ART is initiated (CM-IRIS) [36,37]. Cryptococcal infection tends to affect the central nervous system in the majority of immunocompromised individuals, with poor outcomes irrespective of drug susceptibility [17,38,39]. Clinically, cryptococcosis-associated IRIS presents as a dysregulated proinflammatory immune response, which co-occurs with the increase in CD4+ T cell counts and the reduction in the peripheral blood HIV viral load [37]. Up to twenty percent of patients simultaneously affected by HIV and *Cryptococcus* develop cryptococcosis-associated meningitis IRIS (CM-IRIS), which manifests a few days to a few months after ART initiation, and the mortality averages at 15% [40,41]. It can occur in patients diagnosed with cryptococcal meningitis and in undiagnosed people affected by HIV [42]. Rarely, IRIS presents with other symptoms, such as pulmonary, cutaneous/mucosal, or vascular symptoms [43,44,45]. Thus, there is an urgent need for novel multiomics biomarkers for the prediction and therapeutic prevention of this unfortunate adverse event, particularly on the African continent [46].

### 3.1. CM-IRIS Cellulomics and Proteomics

Multiple cellular alterations have been identified in cryptococcal meningitis IRIS (CM-IRIS), with the specific signature of blood granulocyte activation. In the setting of severe lymphopenia and immunosuppression, ineffective phagocytosis and antigen presentation lead to an increase in fungal burden and HIV viral load [47,48]. An increase in activated circulating neutrophils has been correlated with high mortality and can be considered a predictor of the degree of systemic oxidative stress and fatal outcomes [49].

Several studies have reported that patients with cryptococcal meningitis and late-stage HIV infection accumulate a high cryptococcal antigen burden. Peripheral blood leucocytes from these patients showed a disproportionate cellular increase in CD66^+^ neutrophils and CD16^+/−^ monocytes and a lack of expansion of T-helper type 1 cell populations after the initiation of anti-HIV and antifungal treatments [50,51]. Research studies have reported that the phenotype of mononuclear cells changes in CM-IRIS patients who fail to clear *Cryptococci* from the cerebrospinal fluid (CSF) [52]. There are alterations in the immune cell populations detected in the peripheral blood before the initiation of antiretroviral therapy (ART), such as increased frequencies of activated CD14^+^HLA-DR and CD14^+^CD86^+^ blood monocytes, that have been observed pre-ART. During CM-IRIS episodes, the compartmentalized cerebrospinal fluid monocyte biomarkers shift from the classic (CD14^high^CD16^negative^) to an intermediate/pro-inflammatory phenotype (CD14^high^CD16^dim^) [53]. CD14^dim^CD16^high^ blood monocytes produce high amounts of TNFα and IL6 ex vivo in response to IFNγ stimulation [54].

An additional factor that contributes to the pro-inflammatory state is the inappropriate hyperactivation of macrophages that persistently harbor replicated HIV, especially in patients who develop ART resistance due to the enhanced expression of efflux transporters, such as MRP1 and BCRP [55]. Markers of monocyte activation in plasma, like D-dimer, C-reactive protein, and serum amyloid A, are particularly linked to higher mortality rates [56,57]. These patients also exhibit high intracranial pressure and laboratory markers of neurological damage, as described below. During the inflammatory response, the CD40 ligand (TNFSF5), a marker of endothelial cells, platelets, and tissue macrophages, is converted into a soluble marker after shedding into the bloodstream [58]. High plasma levels of the soluble CD40 ligand were found to counteract the TLR7 and TLR9 receptors and suppress IFNα production, which was observed in CM-IRIS patients [59]. The appearance of soluble forms of sCD163, sCD14, CCL3, and sCD40L in CSF serves as a biomarker of CNS inflammation during CM-IRIS [59,60].

The imbalance of numerous cytokines and chemokines in patients’ blood and CSF can be measured and used to obtain biomarkers of CM-IRIS (e.g., IL6, IL18, TNFα, IL5, IL7, IL17, GCSF, GMCSF, CCL11, and CXCL10) [51,61,62,63]. These cytokines are released into the systemic circulation from inflammatory foci that occur in the brain [63]. The peripheral blood from patients who had fatal outcomes revealed low glucuronoxylomannan (GXM)- and antigen-driven monocyte responses (CD16^low/neg^HLA-DR^low^), significantly reduced TNFα, and increased IL6, IL10, and CXCL10 production [50]. To diminish the cytokine storm during a CM-IRIS event, several biotherapeutics have been proposed and tested as adjunct therapies to mitigate CM-IRIS’s progression, such as CXCR7, which targets receptors on CD14^+^CD16^+^ monocytes [64]. The recently utilized RAG1-deficient mouse model is expected to contribute to a better understanding of C-IRIS’s cellular pathogenesis and aid in the development of targeted therapeutic strategies [65].

The dysregulation of cytokine/receptor pairing also plays a role in CM-IRIS. For example, the augmentation of CSF receptor/ligand pairs CCL2/CXCL10, CXCR3/CCR5 and CCL3/CXCL10 in HIV/CM patients with neurological deterioration leads to the development of CM-IRIS after ART initiation [66]. As these cytokines and chemokines play a role in augmenting the influx of CD4+ T cells and myeloid cells into the CNS and CSF, such interactions contribute to blood–brain barrier damage and increased immuno-neurological inflammation [66]. Macrophage-specific receptor shedding has been reported in the cerebrospinal fluid of patients who are at a higher risk of mortality from CM-IRIS. The IL7/IL7R interactions play a role in T cell survival during inflammatory events and subsequent recovery, and abnormally high IL7 plasma levels are strongly associated with CM-IRIS events [52]. A dysbalanced cytokine milieu affects the differentiation of T-helper 0 cells into the Th1 and Th2 types, which, in turn, impairs the development of an adaptive immune response to *Cryptococcus* spp. [61,67]. The recruitment of naïve T cells to the central nervous system during immune restoration results in a pathological immune response [68]. The expression of immunosuppressive programmed death ligand 1 (PDL1) on CSF T cells, CD56^dim^ monocytes, and CD56^bright^ NK cell subsets is also associated with the immunopathology of CM-IRIS [53].

Proteomics studies have shown the paucity of CSF cytokines to be predictive of CM-IRIS at the time of ART initiation [69]. In patients with late-stage HIV infection, these biomarkers overlap with those shown for fatal cryptococcal meningitis, such as low levels of GCSF, CSF, IFNγ, IL5, and IL6; a high CSF fungal burden; and the presence of CD4^neg^CD8^neg^ T cells [70]. Additionally, CSF proteomics showed that high levels of chemokines MCP-1 (CCL2) and MIP1α (CCL3) were predictive of CM-IRIS [71]. In severe cases of cryptococcal meningitis, the infected macrophages express so-called alternative activation cluster differentiation markers (e.g., CD200, CD163, and CD206) and are unable to eradicate *Cryptococcus* spp. from the CSF, spreading the pathogen to the central nervous system [70,72]. A recent study revealed sex-specific neuroimmune signatures of fatal outcomes in patients who initiated antifungal therapies. Lower levels of CSF, CXCL10, CCL11, IL12p70, TNFα, CD40L, and IL17A are associated with high 18-week mortality in women; higher IL13 and IL15 levels are associated with mortality in men, and decreased levels of IL10 and IL13 with both sex groups [73].

The antibody-producing B cell function was found to be altered in individuals who subsequently developed CM-IRIS. The insufficient production of IgM antibodies in response to the cryptococcal polysaccharide antigens pustulan, laminarin, and GXM correlated with a higher risk of CM-IRIS due to inadequate antibody-mediated cryptococcal antigen clearance [74]. The expression of PD1 on B/plasmablast cells was found to be protective from fatal cryptococcal disease [75]. It would be interesting to assess the expression of PD1/PDL1 on both types of populations, T and B cells, with respect to CM-IRIS outcomes.

The overwhelming majority of patients with advanced HIV infections suffer from long-lasting HIV-associated neurocognitive disorders (HAND), which are exacerbated by cryptococcal meningitis and CM-IRIS [76]. A high percentage of TNFα/IFNγ-expressing and CD8^+^CD107^+^ T cells in the CSF is accompanied by a low degranulation capacity, which is accompanied by increased levels of soluble sCD163 and sCD14. This, most likely, contributes to intracranial inflammation during and after the CM-IRIS event [77]. HIV^+^CD14^+^CD16^+^ monocytes transmigrate across the blood–brain barrier, becoming a source of pro-inflammatory biomarkers and appearing to be the main drivers of the neuro-immunopathology [78,79]. Identifying local and systemic biomarkers attributable to the cellular response to HIV alone and in combination with cryptococcosis can be useful in designing diagnostic assays that can alert clinicians of CM-IRIS in hospital settings. Developing combinatory scoring based on immunomics data and utilizing predictive modeling will soon be possible for clinicians in the estimation of outcomes in HIV patients affected by newly diagnosed cryptococcal meningitis and/or CM-IRIS [80,81]. Combined with clinical risk factors and demographics, immunomics data may be applied to improve screening and subsequent management strategies [41].

### 3.2. Genomics and Transcriptomics of CM-IRIS

The most valuable transcriptomic studies are those that assess longitudinal blood biomarker changes from samples collected before ART initiation and during or after CM-IRIS events. For example, the overexpression of integrins and chemokine transcripts in the peripheral blood was found to precede CM-IRIS events and thus they can be considered as predictive biomarkers [82].

**Figure 2:**
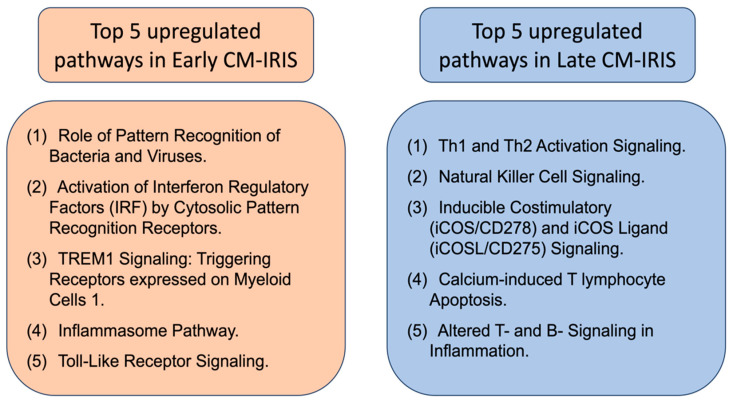
Top 5 canonical pathways that were overrepresented in upregulated transcript sets in early and late (non-fatal) CM-IRIS subgroups. The top five pathways are those with the largest proportion of transcripts upregulated within the pathway (based on the ingenuity pathway analysis assignment of individual transcripts [82]). Data were extracted for review from [82].

Two molecular subtypes of non-fatal CM-IRIS events have been identified through the analysis of peripheral blood leukocyte transcriptomic signatures. The first is an early CM-IRIS event (occurring within two months on ART) and the second is a late CM-IRIS event (occurring after 3 months on ART) [82]. Early CM-IRIS exhibits the upregulation of the innate immune pathways, inflammasome components (NLR receptors 3 and 4, AIM2, CASP1, CASP5), and Toll-like receptors (TLR2, TLR4) [82]. These transcripts can be arranged into five canonical pathways, depicted in Figure 2 (left box), which represent the pathway-based signatures of late cryptococcal immune reconstitution inflammatory syndrome. Late CM-IRIS, in samples collected 12 weeks after ART initiation, showed an increased proportion of gene signatures from adaptive immune cell activation (T helpers Th1 and Th2 activation signaling) and innate natural killer immune biomarkers (Figure 2, right box) [82]. This increase, however, was significantly delayed when compared to the control group (no CM-IRIS), which suggests that the recovery of adaptive immunity (T and B cells) on ART is delayed in the late CM-IRIS group, with sparse functional maturation and quantitative cellular recovery.

This observation is corroborated by a functional study that showed a significant reduction in type II IFNγ production by mononuclear cells in response to stimulation with cryptococcal antigens, as well as by imbalances in innate type I interferons before and during CM-IRIS events [67,83]. Taken together, these data demonstrate the imbalance of the innate and adaptive immune axes in cryptococcal IRIS. 

Recently, we assessed transcriptomic profiles attributable to CM-IRIS and non-IRIS-related outcomes and characterized the changes, comparing the ART-induced gene expression between combined CM/HIV patients who developed CM-IRIS and those who did not [82,84]. Even before ART initiation, the non-fatal CM-IRIS group showed under-expressed transcripts encoding antiviral defense and type I and II interferon proteins, which function to suppress HIV replication, while antimicrobial/ anticryptococcal defense transcripts were overexpressed [82].

Our most recent study specifically focused on fatal CM-IRIS cases. The elevated baseline (pre-ART) expression of interferon-gamma (IFNγ) was identified in patients who died from early CM-IRIS [85]. This suggests that the expression of components of antiviral defense pathways, including type I/II interferons and IFN-induced genes, could provide differentiative biomarkers between fatal and non-fatal CM-IRIS cases [82]. Transcriptomic biomarkers associated with activated granulocytes and complement components, including oxidases, arginase, integrins, etc., were found to precede CM-IRIS events. These biomarkers could potentially be used for the early detection or prediction of fatal CM-IRIS. Fatal CM-IRIS events are characterized by markers of tissue destruction, including interleukin-1 (IL1), interleukin-6 (IL6), Toll-like receptor (TLR), triggering receptor expressed on myeloid cells (TREM), high-mobility group box 1 (HMGB1), inflammasome components, NRF2-mediated pathways, the NF-κB pathway, the p38-MAPK pathway, matrix metalloproteinases (MMPs), and Rho-GDI (Rho GDP-dissociation inhibitor) molecules [82]. Using ingenuity pathway analysis (IPA) software (Qiagen Inc., Redwood City, CA, USA, release 2021) and comparative transcript expression measurements, we identified the pathway-based signature of fatal cryptococcal immune reconstitution inflammatory syndrome (Table 1).

The twenty-three pathways depicted are subdivided into two parts (the upper 14 are upregulated in the fatal CM-IRIS group in comparison to all studied comparator groups, and the lower nine are downregulated in fatal CM-IRIS, in comparison with the same comparator groups of patients).

The hallmark of fatal CM-IRIS is a combination of pathways of innate immune system activation and acute phase response/oxidative stress signaling. Assuming that the same pathways are upregulated in neuronal tissues, such reactive oxygen species-driven oxidative stress can lead to focal neuronal loss in the CNS and subsequent neurodegenerative disease. 

The identification of the optimal timing for ART initiation in late-stage HIV/CM patients has been a focus of clinical research since its proposed definition [86]. The transcriptomic research data, described above, were based on early and deferred ART initiation in reference to antifungal treatment (1 week and 4 weeks, respectively) [85,87]. Of note, there were no fatal CM-IRIS events in the deferred group [87]. Our discovery of the different pathways that drive the early, late, and fatal forms of immune reconstitution inflammatory syndrome in this population contributes to an understanding of these aspects by comparing the survival between the early ART and deferred ART groups. In corroboration with our data, a recent clinical trial conducted in China concluded that deferring ART by 6 weeks or more decreased all-cause mortality, including that from CM-IRIS [88].

Although genome-wide association studies for CM-IRIS susceptibility have not been reported, the genetic-driven susceptibility to cryptococcosis in an HIV patient cohort has recently been identified, with an association with the novel CSF1 gene locus indicating increased odds of developing cryptococcal meningitis [89]. The CD127 (also interleukin-7 receptor subunit alpha) gene T/T allele homozygosity (rs6897932) was associated with faster CD4+ T cell recovery on ART in individuals affected by (in comparison to those with a wild-type C/C allele) [90,91]. This genetic association suggests that the genetic variation in the CD127 gene may play a role in influencing the rate of CD4+ T cell recovery in response to ART. Genetic variants in the TLR1, 2, 6, and 9 genes predispose patients to the development of cryptococcal meningitis and a more severe disease course [92]. It is possible that some patients are predisposed to developing IRIS based on their individual genetic make-up; however, more research needs to be conducted in the field of the genomics of immune reconstitution disorders to support this hypothesis [89,93,94,95]. Understanding the genetic associations is essential for personalized medicine approaches, allowing for tailored interventions and treatment strategies based on an individual’s genetic profile.

## 4. Concluding Remarks and Future Directions

In conclusion, the intricate immunopathology of CM-IRIS unfolds through the dysregulated expression of cellular molecules and extracellular receptors by distinct cellular subsets, particularly monocytes and granulocytes. The consequential production of pro-inflammatory proteins during CM-IRIS plays a pivotal role in shaping the outcomes in terms of an ART-associated immune reconstitution pathology, eliciting discernible adverse host reactions. Notably, the molecular underpinnings of CM-IRIS lack absolute distinctiveness, exhibiting shared features with other IRIS manifestations, such as tuberculosis IRIS, fatal cryptococcal meningitis, and acute respiratory distress syndrome (ARDS) caused by other viral infections [12,82,96,97]. The transcriptomic congruences between fatal ARDS and CM-IRIS encompass key pathways, including HMGB1, oxidative stress response transcripts, inflammasome activation, and Toll-like receptor signaling [97].

The phenomenon of IRIS-like inflammatory reactions has been described for other conditions in immunocompromised and non-immunocompromised patients [48]. The parallel activation of the innate immune and oxidative stress pathways in SARS-CoV-2, an RNA virus, reflects the mechanisms induced by HIV, underscoring intriguing similarities in the context of fatal acute respiratory distress syndrome (ARDS) and fatal CM-IRIS [98]. Furthermore, the administration of mRNA vaccines for newly emerged infectious diseases may precipitate type I/II interferon production by lymphocytes and monocyte/macrophages, which highlights the possibility of inadvertently inciting an inflammatory reaction akin to IRIS [99]. Thus, it is important to continue studying the IRIS-like inflammation, expanding the omics portfolio to include genomics, metabolomics, and, moreover, dual host–pathogen RNA-sequencing methodologies [100,101]. In the pursuit of a comprehensive understanding of immune reconstitution inflammatory syndrome, a reliance on high-throughput laboratory methodologies in understanding antimicrobial resistance, computer-aided drug design, and the discovery of antimicrobial nanomaterials are imperative [1,102,103,104]. Since IRIS-like inflammation is largely driven by microbial antigens, there has been an ever-increasing need for novel machine learning models to predict neutralizing therapeutic peptides that would lessen immune response [105]. The integration of IRIS multiomics data modeling and predictive analytics emerges as indispensable in formulating evidence-based solutions to the multifaceted challenges encountered in the clinical, biomedical, and public health domains [100,106].

## Figures and Tables

**Figure 1:**
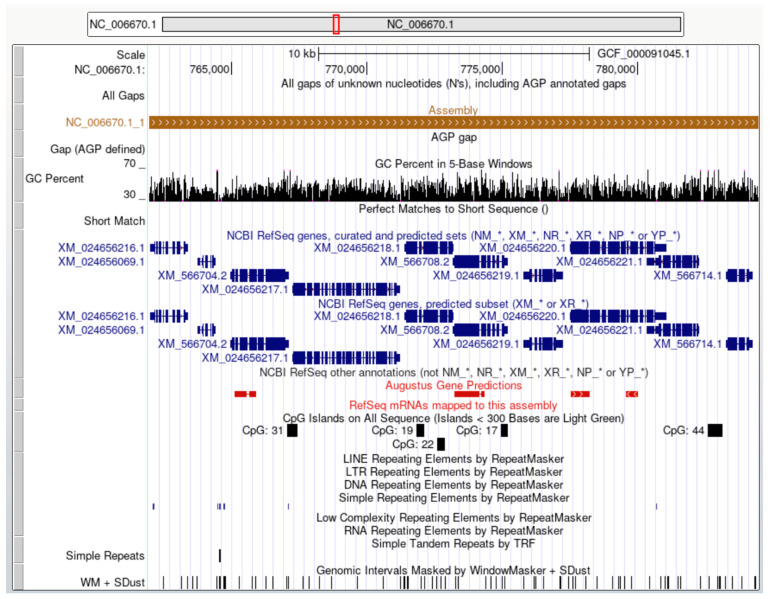
UCSC Genome Browser view of basidiomycetes *C. neoformans* var. *neoformans* JEC21 (GCF_000091045.1). View Link (accessed 1 February 2024). The following tracks are shown (top to bottom): genome base positions; all gaps; assembly NC_006670.1:761,973-784,475; GC Percent in 5-Base Windows; Perfect Match to Short Sequence; RefSeq gene predictions from NCBI (shown in dark blue); Augustus Gene Predictions (shown in red); CpG islands track; Repeating Elements by RepeatMasker; Simple Tandem Repeats by TRF; and Genomic Intervals Masked by WindowMasker + SDust track.

**Table 1:** Comparisons of the relative degree of expression of immune genes and immune/inflammatory pathways between fatal CM-IRIS and comparator/control groups.

Molecular Pathways	Column 2. Fatal CM-IRIS Group (Event) versus Fatal CM-IRIS	Column 3. Fatal CM-IRIS Group (Event) versus No CM-IRIS Group or Death from Meningitis (Control)	Column 4. Fatal CM-IRIS Group (Event) versus No CM-IRIS or Death from Meningitis Group	Column 5. Fatal CM-IRIS Group (Event) versus CM-IRIS Survivor Group	Column 6. Fatal CM-IRIS Group (Event) versus Death from Meningitis Group
TREM1 Signaling	↑↑	-	↑↑↑	-	↑↑↑
Toll-Like Receptor Signaling	↑↑	↑↑↑	↑↑↑	↑	↑↑↑
Acute Phase Response Signaling	↑↑	↑↑↑	↑↑↑	↑	↑↑↑
Inflammasome Pathway	↑	↑↑↑	↑↑↑	-	↑↑
Fcγ Receptor-Mediated Phagocytosis in Macrophages	-	↑↑↑	↑↑↑	-	↑↑
Interferon Signaling	-	↑↑	↑↑	-	↑↑
LPS/IL1-Mediated Inhibition of RXR function	↑↑	↑↑	↑	↑↑↑	↑↑
PD-1, PD-L1 Immunotherapy Pathway	↑↑↑	↑↑↑	↑↑	↑↑↑	↑↑
IL6 Signaling	↑	-	↑↑↑	↑↑	↑↑
P38 MAPK Signaling	↑↑	↑↑↑	↑↑	↑↑	↑
Rho-GDI Signaling	↑↑	↓↓	-	-	↑
Role of Pattern Recognition	↑	↑↑	↑↑↑		↑
HMGB1 Signaling	-	↑	↑↑	-	-
T Cell Exhaustion Signaling Pathway	↑	-	-	-	-
NRF2-Mediated Oxidative Stress Response	↓	↑	↑	↓	↓
NF-kB Activation by Viruses	↓↓↓	↓↓	-	↓	↓
Signaling by Rho Family of GTPases	↓↓	↑↑↑	↑↑	-	↓
Th1 Pathway	-	-	-	↓↓↓	↓↓
Complement System	-	-	-	-	↓↓
eNOS Pathway	↓↓↓	-	-	↓↓	↓↓
fLMP Signaling in Neutrophils	↓↓↓	-	-	↓↓	↓↓
CD28 Signaling in T-Helper Cells	↓↓↓	-	↓↓	↓↓↓	↓↓
Oxidative Phosphorylation	-	-	-	-	↓↓↓

↑, ↓; ↑↑, ↓↓; ↑↑↑, ↓↓↓ arrows represent overall degree of up- or downregulation for immune pathways, based on the enrichment z-scores for genes that were significantly up- or downregulated within the molecular pathway (**Column 1**). The degree in each comparator group is relative to Fatal CM-IRIS group (**Column 2**) [85]. **Column 2.** Fatal CM-IRIS group (event) versus fatal CM-IRIS group at 1 week post-ART (most proximal time point to the event). **Column 3**. Fatal CM-IRIS group (event) versus no CM-IRIS group or death from meningitis group (control group) at 1 week post-ART. **Column 4.** Fatal CM-IRIS group (event) versus no CM-IRIS or death from meningitis group at week 4 post-ART. **Column 5.** Fatal CM-IRIS group (event) versus CM-IRIS survivor groups (event). **Column 6.** Fatal CM-IRIS group (event) versus death from meningitis group at week 1 post-ART. Adopted from [85] with the author’s permission under common copyrights.
